# 2561. Population Pharmacokinetic Analyses for Ceftobiprole Using Data from Phase 1 and 3 Studies

**DOI:** 10.1093/ofid/ofad500.2178

**Published:** 2023-11-27

**Authors:** Anthony P Cammarata, Karine Litherland, M Courtney Safir, Sujata M Bhavnani, Mikael Saulay, Jennifer Smart, Mark E Jones, Marc Engelhardt, Christopher M Rubino

**Affiliations:** Institute for Clinical Pharmacodynamics, Schenectady, New York; Basilea Pharmaceutica International Ltd, Allschwil, Basel-Landschaft, Switzerland; Institute for Clinical Pharmacodynamics, Schenectady, New York; Institute for Clinical Pharmacodynamics, Schenectady, New York; Basilea Pharmaceutica International Ltd, Allschwil, Basel-Landschaft, Switzerland; Basilea Pharmaceutica International Ltd, Allschwil, Basel-Landschaft, Switzerland; Basilea Pharmaceutica International Ltd., Allschwil, Switzerland, Allschwil, Basel-Landschaft, Switzerland; Basilea Pharmaceutica International Ltd., Allschwil, Switzerland, Allschwil, Basel-Landschaft, Switzerland; Institute for Clinical Pharmacodynamics, Schenectady, New York

## Abstract

**Background:**

Ceftobiprole medocaril is an intravenously administered cephalosporin prodrug that is rapidly converted *in vivo* to ceftobiprole which has activity against Gram-positive and -negative organisms. A population pharmacokinetic (PK) model was constructed from nine Phase 1 studies and three Phase 3 studies in patients with acute bacterial skin and skin structure infections (ABSSSI) or hospital- or community-acquired bacterial pneumonia (HABP or CABP) and refined using Phase 3 data from patients with *Staphylococcus aureus* bacteremia (SAB).

**Methods:**

Structural PK models for systemic compartments and linear vs. non-linear elimination were considered. Covariate analyses were carried out. Model evaluation involved goodness-of-fit and prediction-corrected visual predictive check plots and a sampling-importance-resampling procedure. The model was externally qualified and refined using Phase 3 SAB PK data.

**Results:**

The initial dataset included 4890 plasma concentrations from 773 healthy subjects or patients with ABSSSI, HABP, or CABP. This dataset was expanded to include 1134 plasma concentrations from 180 patients with SAB. External qualification showed that the model predicted data from patients with SAB well but the model was refined to best capture data from these patients. The refined model (three-compartment model with linear elimination) provided a robust fit to the pooled data (**Figure 1**). Model-based simulations to predict exposure in subgroups (i.e., renal function, body size, sex, and infection type) showed that renal function is the most clinically relevant covariate, consistent with the predominance of renal elimination for ceftobiprole (**Figure 2**). While model-based simulations showed that predicted exposure in subjects with extreme body weight (120 kg) fall below the bioequivalence reference range of healthy subjects weighing 75 kg, these weight-based differences are unlikely to be clinically significant. Neither sex nor infection type are expected to have a clinically relevant impact.

**Figure:**
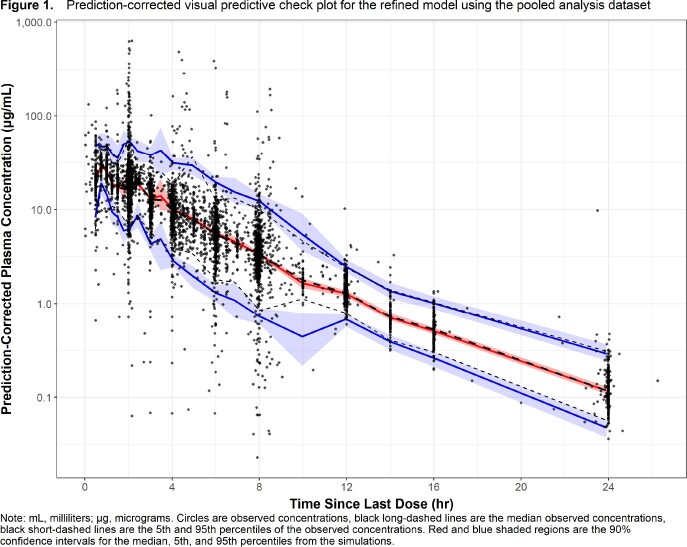

**Figure:**
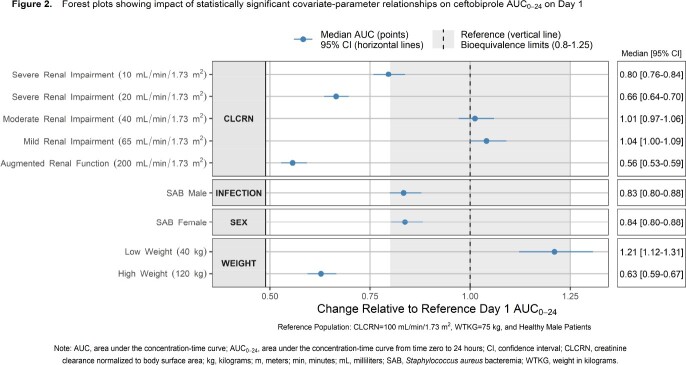

**Conclusion:**

A population PK model that described the plasma PK of ceftobiprole well in healthy and infected patients was developed. The derived measures of plasma exposure are expected to be both accurate and precise.

**Disclosures:**

**Anthony P. Cammarata, M.S.**, Adagio Therapeutics, Inc.: Grant/Research Support|Albany Medical Center: Grant/Research Support|Amplyx Pharmaceuticals, Inc.: Grant/Research Support|AN2 Therapeutics: Grant/Research Support|Antabio SAS: Grant/Research Support|Arcutis Biotherapeutics, Inc.: Grant/Research Support|B. Braun Medical Inc.: Grant/Research Support|Basilea Pharmaceutica: Grant/Research Support|BioFire Diagnostics LLC: Grant/Research Support|Boston Pharmaceuticals: Grant/Research Support|Cidara Therapeutics Inc.: Grant/Research Support|Cipla USA: Grant/Research Support|Crestone Inc.: Grant/Research Support|CXC: Grant/Research Support|Debiopharm International SA: Grant/Research Support|Entasis Therapeutics: Grant/Research Support|Genentech: Grant/Research Support|GlaxoSmithKline: Grant/Research Support|Hoffmann-La Roche: Grant/Research Support|ICPD: Employee|Inotrem: Grant/Research Support|Insmed Inc.: Grant/Research Support|Iterum Therapeutics Limited: Grant/Research Support|Kaizen Bioscience, Co.: Grant/Research Support|KBP Biosciences USA: Grant/Research Support|Matinas Biopharma: Grant/Research Support|Meiji Seika Pharma Co., Ltd.: Grant/Research Support|Melinta Therapeutics: Grant/Research Support|Menarini Ricerche S.p.A.: Grant/Research Support|Mutabilis: Grant/Research Support|Nabriva Therapeutics AG: Grant/Research Support|Paratek Pharmaceuticals, Inc.: Grant/Research Support|Qpex Biopharma: Grant/Research Support|Sfunga Therapeutics: Grant/Research Support|Spero Therapeutics: Grant/Research Support|Suzhou Sinovent Pharmaceuticals Co.: Grant/Research Support|Theravance: Grant/Research Support|tranScrip Partners: Grant/Research Support|University of Wisconsin: Grant/Research Support|Utility Therapeutics: Grant/Research Support|ValanBio Therapeutics Inc.: Grant/Research Support|VenatoRx: Grant/Research Support **Karine Litherland, Ph.D.**, Basilea Pharmaceutica International Ltd, Allschwil, Switzerland: Full time employee|Basilea Pharmaceutica International Ltd, Allschwil, Switzerland: Stocks/Bonds **M. Courtney Safir, PharmD**, Adagio Therapeutics, Inc.: Grant/Research Support|Albany Medical Center: Grant/Research Support|Amplyx Pharmaceuticals, Inc.: Grant/Research Support|AN2 Therapeutics: Grant/Research Support|Antabio SAS: Grant/Research Support|Arcutis Biotherapeutics, Inc.: Grant/Research Support|B. Braun Medical Inc.: Grant/Research Support|Basilea Pharmaceutica: Grant/Research Support|Biofire Diagnostics LLC: Grant/Research Support|Boston Pharmaceuticals: Grant/Research Support|Cidara Therapeutics Inc.: Grant/Research Support|Cipla USA: Grant/Research Support|Crestone Inc.: Grant/Research Support|CXC: Grant/Research Support|Debiopharm International SA: Grant/Research Support|Entasis Therapeutics: Grant/Research Support|Genetech: Grant/Research Support|GlaxoSmithKline: Grant/Research Support|Hoffmann-La Roche: Grant/Research Support|ICPD: Employee|Inotrem: Grant/Research Support|Insmed Inc.: Grant/Research Support|Iterum Therapeutics Limited: Grant/Research Support|Kaizen Bioscience, Co.: Grant/Research Support|KBP Biosciences USA: Grant/Research Support|Matinas Biopharma: Grant/Research Support|Meiji Seika Pharma Co., Ltd.: Grant/Research Support|Melinta Therapeutics: Grant/Research Support|Menarini Ricerche S.p.A.: Grant/Research Support|Mutabilis: Grant/Research Support|Nabriva Therapeutics AG: Grant/Research Support|Paratek Pharmaceuticals, Inc.: Grant/Research Support|Qpex Biopharma: Grant/Research Support|Sfunga Therapeutics: Grant/Research Support|Spero Therapeutics: Grant/Research Support|Suzhou Sinovent Pharmaceuticals Co.: Grant/Research Support|Theravance: Grant/Research Support|tranScrip Partners: Grant/Research Support|University of Wisconsin: Grant/Research Support|Utility Therapeutics: Grant/Research Support|ValanBio Therapeutics, Inc.: Grant/Research Support **Sujata M. Bhavnani, PharmD; MS; FIDSA**, Adagio Therapeutics, Inc.: Grant/Research Support|Albany Medical Center: Grant/Research Support|Amplyx Pharmaceuticals, Inc.: Grant/Research Support|AN2 Therapeutics: Grant/Research Support|Antabio SAS: Grant/Research Support|Arcutis Biotherapeutics, Inc.: Grant/Research Support|B. Braun Medical Inc.: Grant/Research Support|Basilea Pharmaceutica: Grant/Research Support|BioFire Diagnostics LLC: Grant/Research Support|Boston Pharmaceuticals: Grant/Research Support|Cidara Therapeutics Inc.: Grant/Research Support|Cipla USA: Grant/Research Support|Crestone Inc.: Grant/Research Support|CXC: Grant/Research Support|Debiopharm International SA: Grant/Research Support|Entasis Therapeutics: Grant/Research Support|Genentech: Grant/Research Support|GlaxoSmithKline: Grant/Research Support|Hoffmann-La Roche: Grant/Research Support|ICPD: Ownership Interest|Inotrem: Grant/Research Support|Insmed Inc.: Grant/Research Support|Iterum Therapeutics Limited: Grant/Research Support|Kaizen Bioscience, Co.: Grant/Research Support|KBP Biosciences USA: Grant/Research Support|Matinas Biopharma: Grant/Research Support|Meiji Seika Pharma Co., Ltd.: Grant/Research Support|Melinta Therapeutics: Grant/Research Support|Menarini Ricerche S.p.A.: Grant/Research Support|Mutabilis: Grant/Research Support|Nabriva Therapeutics AG: Grant/Research Support|Paratek Pharmaceuticals, Inc.: Grant/Research Support|Qpex Biopharma: Grant/Research Support|Sfunga Therapeutics: Grant/Research Support|Spero Therapeutics: Grant/Research Support|Suzhou Sinovent Pharmaceuticals Co.: Grant/Research Support|Theravance: Grant/Research Support|tranScrip Partners: Grant/Research Support|University of Wisconsin: Grant/Research Support|Utility Therapeutics: Grant/Research Support|ValanBio Therapeutics Inc.: Grant/Research Support|VenatoRx: Grant/Research Support **Mikael Saulay, MSc**, Basilea Pharmaceutica International Ltd, Allschwil, Switzerland: Full time employee|Basilea Pharmaceutica International Ltd, Allschwil, Switzerland: Stocks/Bonds **Jennifer Smart, PhD**, Basilea Pharmaceutica International Ltd, Allschwil, Switzerland: Stocks/Bonds **Mark E. Jones, PhD**, Astellas Pharma Global Development, Inc: Support for the present publication|Basilea Pharmaceutica International Ltd: Employee of Basilea Pharmaceutica International Ltd|Basilea Pharmaceutica International Ltd: Stocks/Bonds **Marc Engelhardt, MD**, Astellas Pharma Global Development, Inc.: Support for the present publication|Basilea Pharmaceutica International Ltd: Employee of Basilea Pharmaceutica International Ltd|Basilea Pharmaceutica International Ltd: Stocks/Bonds **Christopher M. Rubino, PharmD**, Adagio Therapeutics, Inc.: Grant/Research Support|Albany Medical Center: Grant/Research Support|Amplyx Pharmaceuticals, Inc.: Grant/Research Support|AN2 Therapeutics: Grant/Research Support|Antabio SAS: Grant/Research Support|Arcutis Biotherapeutics, Inc.: Grant/Research Support|B. Braun Medical Inc.: Grant/Research Support|Basilea Pharmaceutica: Grant/Research Support|BioFire Diagnostics LLC: Grant/Research Support|Boston Pharmaceuticals: Grant/Research Support|Cidara Therapeutics Inc.: Grant/Research Support|Cipla USA: Grant/Research Support|Crestone Inc.: Grant/Research Support|CXC: Grant/Research Support|Debiopharm International SA: Grant/Research Support|Entasis Therapeutics: Grant/Research Support|Genentech: Grant/Research Support|GlaxoSmithKline: Grant/Research Support|Hoffmann-La Roche: Grant/Research Support|ICPD: Ownership Interest|Inotrem: Grant/Research Support|Insmed Inc.: Grant/Research Support|Iterum Therapeutics Limited: Grant/Research Support|Kaizen Bioscience, Co.: Grant/Research Support|KBP Biosciences USA: Grant/Research Support|Matinas Biopharma: Grant/Research Support|Meiji Seika Pharma Co., Ltd.: Grant/Research Support|Melinta Therapeutics: Grant/Research Support|Menarini Ricerche S.p.A.: Grant/Research Support|Mutabilis: Grant/Research Support|Nabriva Therapeutics AG: Grant/Research Support|Paratek Pharmaceuticals, Inc.: Grant/Research Support|Qpex Biopharma: Grant/Research Support|Sfunga Therapeutics: Grant/Research Support|Spero Therapeutics: Grant/Research Support|Suzhou Sinovent Pharmaceuticals Co.: Grant/Research Support|Theravance: Grant/Research Support|tranScrip Partners: Grant/Research Support|University of Wisconsin: Grant/Research Support|Utility Therapeutics: Grant/Research Support|ValanBio Therapeutics Inc.: Grant/Research Support|VenatoRx: Grant/Research Support

